# Ethanolic Extracts of *Cissus quadrangularis* Linn. (Vitaceae) Attenuate Vincristine-Induced Peripheral Neuropathy in Rats: An Evidence of the Antioxidant, Calcium Inhibitory, and Neuromodulatory Properties

**DOI:** 10.1155/adpp/8822369

**Published:** 2024-12-12

**Authors:** Feigni Youyi Marcelle Olga, Mbiantcha Marius, Yousseu Nana William, Tsafack Eric Gonzal, Djuichou Nguemnang Stephanie Flore, Noungoua Mbeugangkeng Chrétien, Atsafack Mboudem Lylie Gisèle, Ateufack Gilbert

**Affiliations:** ^1^Laboratory of Animal Physiology and Phytopharmacology, Department of Animal Biology, Faculty of Science, University of Dschang, Dschang, Cameroon; ^2^Laboratory of Biology and Physiology of Animal Organisms, Department of Biology of Animal Organisms, Faculty of Science, The University of Douala Cameroon, P.O. Box 24157, Douala, Cameroon

**Keywords:** 5-HT, antioxidant, *Cissus quadrangularis*, GABA, neuropathic pain, total calcium, vincristine

## Abstract

*Cissus quadrangularis* Linn. (*C. quadrangularis*, Vitaceae) is a plant reported to treat injured tendons, broken bones, asthma, stomach ache, scurvy, and digestive disorders. The present study evaluated the antihyperalgesic effects of ethanolic extract of *C. quadrangularis* Linn. Vincristine sulfate (100 μg/kg, i.p.) was administered in rats for 10 days with 2 days break to induce painful peripheral neuropathy. Mechanical hyperalgesia and allodynia tests were performed to assess the threshold of painful neuropathy. Calcium levels in the sciatic nerve, oxidant stress markers, and levels of GABA and 5-HT were also determined in the brain and spinal cord after 15 days. Ethanolic extract of *C. quadrangularis* (180 and 360 mg/kg) and pregabalin (50 mg/kg) were administered for 15 consecutive days. The results revealed that the extract significantly (*p* < 0.001) inhibited hyperalgesia and allodynia in animals after vincristine administration. The extract decreased total calcium levels in the sciatic nerve, MDA levels while increasing GSH activity, 5-HT level, as well as GABA levels in the brain and spinal cord. The results of this study suggest that the ethanolic extract of *C. quadrangularis* uses antioxidant capacity, calcium inhibitory action, and neuromodulation of GABA and 5-HT to prevent the development of painful neuropathy after vincristine administration. This demonstrates that *C. quadrangularis* is a promising molecule for the management of peripheral neuropathic pain induced by anticancer drugs.

## 1. Introduction

Pain, known as the most common manifestation of anyone undergoing primary care, also known as one of the world's most debilitating problems, causes a feeling that ranges from mild discomfort to extreme pain, which can be in a well-localized wound and/or be diffuse [1]. Known as a sensory experience associated with an unpleasant emotion, pain is subdivided into nociceptive and neuropathic. Neuropathic pain, which is chronic, has a complex mechanism that can be caused by ischemia, immune disorders, metabolic disorders, infections, toxins, and hereditary and traumatic disorders [2]. Chronic pain in general affects nearly 30% of the world's population [3], while chronic neuropathic pain affects about 3%–17% of the world's population [4]; furthermore, in addition to be a public health problem, neuropathic pain is a real economic and social burden. In affected adults, there is a cessation of activities, absenteeism, early retirement, a drop in productivity, and an increase in healthcare accompanied by very negative social and economic impacts [5]. Although acute pain plays a protective role, chronic pain is of no physiological interest since its chronicity causes depression, sleep disturbances, lethargy, and hyperalgesia [6].

One of the most common neuropathic pains is peripheral neuropathy, which represents 1%–7% in the general population and which is usually caused by taking anticancer drugs (cisplatin, paclitaxel, and vincristine) [7]. Vincristine is a widely used cancer treatment to relieve people with cervix, breast, head, uterus, and neck cancers [8]. However, prolonged use of this drug causes neuropathy with numbness, tingling, and burning pain [9]. In fact, vincristine has the capacity to fix the *β*-tubulins of microtubules, which leads to the arrest of its mitotic divisions, and degeneration of the neurons of the peripheral nerves follows [10]. Subsequently, at the peripheral level, there is the activation of immune cells such as macrophages, T cells, neutrophils, dendritic cells, and natural killer cells, which have been shown to play a role in the development and maintenance of neuropathic pain. The activation of its cells will result in the production of numerous proinflammatory cytokines and reactive oxygen species, which cause the release of neurotransmitters (glutamate and substance *P*) and adenosine triphosphate in the central and peripheric nerve systems [11]. Moreover, nerve damage leads to the alteration of the electrical properties of the sensory nerve, and the balance between central excitatory and inhibitory signaling is impaired. The neuromodulators such as serotonin (5-HT) released in the peripheric and central nerve systems contribute to nociceptive sensitization and maintenance of neuropathic pain through 5-HT_3_, 5-HT_4_, and 5-HT_6_ in the spinal cord. In addition, inhibitory interneurons release less of *γ*-aminobutyric acid (GABA), and this drop in GABA can also be caused by hyperexcitability [12]. Generally, the treatment of neuropathic pain relies on a combination of analgesics and many classes of other drugs, which unfortunately cause renal failure, gastric ulceration, coronary artery disease, gastrointestinal bleeding, and thrombotic cardiovascular events [13]. Scientific progress has allowed the understanding of the characteristics of chronic neuropathic pain, as well as the knowledge and understanding of their molecular and cellular mechanisms. This knowledge has led to the development of new therapies for the management of this type of pain. Thus, the use of plants has become an essential alternative medicine for diseases resistant to modern drugs [14].

*Cissus quadrangularis* Linn. (*C. quadrangularis*, Vitaceae), a creeping shrub with a thick quadrangular, fleshy, fibrous, and smooth stem, with four winged internodes [15], is an edible plant found in the hottest regions of India, Sri Lanka, Asia, and Africa [16]. In traditional medicine, this plant is used to treat injured tendons, broken bones, injured ligaments, asthma, stomachache, scurvy, gout, digestive disorders, and as an aphrodisiac [17]. The stem and roots of this plant are known for their antimicrobial, antioxidant, anthelmintic, digestive, analgesic, or spine and back pain activities [18]. Previous studies with *C. quadrangularis* showed its analgesic [19], anti-inflammatory [20], antiosteoporotic [21], and antioxidant [22] properties; moreover its [23] antibacterial, vermifuge, antihemorrhoidal, and analgesic effects [24] on the skeletal system have also been demonstrated. In addition, extracts of this plant have demonstrated antiviral and antifree radical properties [25]. On the other hand, ethyl ethanoate extracts of *C. quadrangularis* suppress nuclear factor kappa B (NF-*κ*B) activation and heme oxygenase-1 stimulation [26]. In addition, many biologically active compounds have already been isolated from *C. quadrangularis*, including hexadecanoic acid, stilbene glucoside trans-resveratrol-3-O-glucoside [16], *β*-sitosterol, *δ*-amyrone, *δ*-amyrin [27], 3 resveratrol, and *β*-sitosterol [28]. The aerial parts of this plant contain 7-oxoonocer-8-ene-3*β* 21 *α* diol [29], 4-hydroxy-2 -methyl-tricos-2-en-22-one, 9-methyloctadec-9-ene, heptadecyl octadecanoate, icosanilico-sanoate, 31-methyl tritiacontan-1-ol, 7-hydroxy-20-oxodocosanyl cyclohexane, l 31-methyl tritiacontanoic acid, taraxerol, isopentacosanoic acid, friedelan-3-one, and 3.3′,4.4′-tetrahydroxybiphenyl [19]. This study was undertaken to priorly characterize the ethanolic extracts of *C. quadrangularis* Linn. (Vitaceae) by LC-MS analysis and evaluate its ability to reduce vincristine-induced neuropathic pain in rats through their antioxidant, calcium inhibitory, and neuromodulatory properties.

## 2. Materials and Methods

### 2.1. Reagents and Equipment

Vincristine sulfate, phosphotungstic acid (H_3_PW_12_O_40_), phosphomolybdic acid (H_3_PMo_12_O_40_), calcium arsenazo III, chlorhydric acid (HCl), Folin–Ciocalteu's reagent, O-phthaldialdehyde (OPT), ninhydrin (0.14 M), carbonate bicarbonate (0.5 M, *pH* 9.9), 10% (v/v) concentrated glacial trichloroacetic acid (TCA), glutamate (0.015 mg/μL), copper tartrate (2.229 mg/mL), carbonate buffer (0.05 M; pH = 10.2), 1% TCA (*v*/*v*), Griess reagent, NaNO_2_, Na_2_HPO_4_. 2H_2_O (0.3 M), dithiobis nitrobenzoate, and phosphate-buffered saline (PBS) were obtained from Sigma-Aldrich. To assess neuropathic pain, mechanical hyperalgesia (analgesimeter, UGO Basil, Italy) and mechanical allodynia (Von Frey filaments) devices were used.

### 2.2. Plant Material and Extraction

The aerial parts of *C. quadrangularis* were harvested in June 2001 in the Littoral region (Moungo department, Nkongsamba, Cameroon). Authentication was carried out at the National Herbarium of Yaoundé in Cameroon in comparison to a sample kept under number 36966 HNC/Cam. After harvesting, the plant was dried in the shade and ground (electric grinder). 300 g of the obtained powder was soaked in 3.25 L of ethanol (99%) and macerated for 48 h and filtered, and the filtrate was concentrated in a rotary evaporator at 70°C, which led to obtaining the ethanolic extract with a yield of 3%.

### 2.3. Quantitative Phytochemistry

#### 2.3.1. Quantification of Total Phenols

A mixture (phosphomolybdic acid [H_3_PMo_12_O_40_] and phosphotungstic acid [H_3_PW_12_O_40_]) is reduced to a solution (molybdenum blue and tungsten), so the absorption varies depending on the quantitative/qualitative composition, of the pH and the phenolic mixtures are obtained by adding sodium carbonate [30]. For this test, a solution of the extract (20 μL, 2 mg/mL) or distilled water (white tube), Folin–Ciocalteu reagent (100 μL, diluted 10 times), and sodium carbonate (80 μL, 20% (*v*/*v*)) was stirred, incubated (water bath, 20°C, 30 min), and the absorbance was read (spectrophotometer, 765 nm). Gallic acid (0.015–2 mg/mL) was used to draw the standard curve which made it possible to obtain the results in milligram equivalent of gallic acid per gram of powder [31].

#### 2.3.2. Quantification of Total Flavonoids

A solution made up of a mixture of the extract (100 μL, 2 mg/mL), aluminum chloride (50 μL, 1.2%), and potassium acetate (50 μL, 120 mM) was incubated (30 min, room temperature) and the optical density was read (415 nm). Quercetin (0.015–2 mg/mL) was used to draw the standard curve which made it possible to obtain the results in milligram equivalent of quercetin per gram of powder [32].

#### 2.3.3. Quantification of Total Tannins

A solution of extract (100 μL, 2 mg/mL) or distilled water (white tube), Folin–Ciocalteu reagent (500 μL, diluted 10 times), sodium carbonate (1000 μL, 35%), and distilled water (8.4 mL) was stirred, incubated (30 min, room temperature), and then the optical density was read at 700 nm. Tannic acid (100, 200, 300, 400, and 500 μg/mL) was used to draw the standard curve used to obtain the results in milligram equivalent of tannic acid per gram of powder [33].

### 2.4. LC-MS Analysis

UHPLCMS analysis in a standardization trial was used to carry out the phytochemistry of the extract. High-resolution mass spectra of the extract were obtained through a spectrometer (QTOF Bruker, Germany) equipped with a HESI source. The spectrometer operates in positive mode (mass range: 100–1500, with a scan rate of 1.00 Hz) with automatic gain control to provide high-accuracy mass measurements within 0.40 ppm deviation using Na formate as a calibrant. Spray voltage of 4.5 kV, capillary temperature of 200°C, and nitrogen used as sheath gas (10 L/min) were parameters used for experiments. An UltiMate 3000 UHPLC system (Thermo Fisher, Germany) equipped with an LC pump, a diode array detector (DAD) (*λ*: 190–600 nm), an autosampler (injection volume: 10 μL), and a column oven (40°C), was connected to the spectrometer. The Synergi MAX-RP 100 A (50 × 2 mm, 2.5 μ particle size) composed of a H_2_O (+0.1% HCOOH) (A)/acetonitrile (+0.1% HCOOH) (B) gradient (flow rate 500 μL/min, injection volume 5 μL) allowed separations. The subsequent gradient program was implemented as follows: the gradient program comprised an isocratic phase of 1.5 minutes at 95% A, followed by a linear gradient up to 100% B for 6 minutes. Upon reaching 100% B, the system returned to its original state (90% A) within a single minute and was allowed to reach equilibrium for a further minute. This enabled the analysis of the sample in question to be conducted.

### 2.5. Neuropathic Pain, Animal Treatment, Induction, and Behavioral Assessment

Thirty six Wistar rats (male and female) weighing between 160 and 200 g (3-4 months) and reared under normal conditions (temperature: 19°C–23°C, 12 h light) at the Research Unit of Animal Physiology and Phytopharmacology of the Department of Animal Biology of the University of Dschang (Cameroon) were divided into 6 groups of 6 rats each. These animals were treated as follows: Group 1 (normal control) was treated with distilled water, Group 2 (negative control) was treated with distilled water (1 mL/100 g of body weight), Group 3 (positive control) was treated with pregabalin (50 mg/kg), and Groups 4, 5, and 6 (tests) were treated with the ethanolic extract of *C. quadrangularis* at respective doses of 90, 180, and 360 mg/kg. All treatments were administered orally on the first day before the start of induction. After administration of the various treatments, an intraperitoneal injection of 100 μg/kg of vincristine sulfate was administered to the rats (except the animals in Group 1) for two series of five days (1^st^, 2^nd^, 3^rd^, 4^th^, and 5^th^ day, then 8^th^, 9^th^, 10^th^, 11^th^, and 12^th^ day) of successive work with 2 days off. After the start of induction, the different treatments were administered daily until the 15^th^ day [34, 35].

For behavioral tests, mechanical hyperalgesia was induced by placing the left hind paw of each rat on an analgesimeter (Ugo Basile) on days 0, 1, 3, 5, 7, 9, 11, 13, and 15; the pain was caused by increasing the pressure (cut-of 250 g) until the paw was withdrawn; the value of the nociceptive threshold was considered as the force (g) obtained [35], while mechanical allodynia was performed using Von Frey filaments. The animals were first placed individually in cages for acclimatization over a period of 30 min before taking the values. Subsequently, these animals gradually received the Von Frey filaments in order of masses perpendicular to their right plantar arches. The pressure was exerted for 5 s until the filament bent. One test consisted of applying the same filament three times in a row for a period of 1 min. The animal's response was considered a painful reaction if it lifted its paw, lifted its leg, or moved its body and licked or bit the stimulated paw or spread the toes. The mass of the filament to which the animal responded was considered the threshold intensity.

### 2.6. Biochemical Analysis

On the 15^th^ day after taking the last behavioral values, all animals were sacrificed. The sciatic nerve, spinal cord, and brain were removed and weighed using a sensitive balance (ADA); the spinal cord was then ground in a solution consisting of 850 μL of 37% HCl and 1 L of butanol, with a ratio of 5 mg per 100 μL of the solution. The whole was centrifuged at room temperature (2000 rpm, 10 min) with a centrifuge (Eppendorf 5804 R, Hamburg), and 500 μL of the supernatant was collected and introduced into a tube containing 1250 μL of heptane and 155 μL of HCl 0.1 M. The whole batch was shaken vigorously for 10 min with a magnetic stirrer (Agimatic REV TFT) and then centrifuged again for 10 min (2000 rpm); only the lower aqueous phase was kept for the 5-HT assay. The sciatic nerve, brain, and other parts of the spinal cord were also ground in 10% PBS (1 g per 10 mL) and centrifuged at room temperature (3000 rpm, 15 min). The supernatants were collected to determine the level of calcium ion (Ca^2+^) in the sciatic nerve, serotonin (5-HT) in the spinal cord, GABA in the brain, nitric oxide (NO), malondialdehyde (MDA), and glutathione (GHS) in the brain and spinal cord.

### 2.7. Total Calcium Level

Calcium arsenazo III reagent has been used for the determination of total calcium in the sciatic nerve [36]. In brief, 15 μL of the sample or standard solution was introduced into dry tubes. PBS was also taken at 15 μL and introduced into the white tubes. Then, 10 μL of Arsenazo III reagent was added and the whole set was incubated for 5 min in a marine bath at 37°C. Spectrophotometer readings were then taken at 650 nm wavelength. The concentration of calcium was expressed as mg/dL.

### 2.8. GHS Concentration

Glutathione was measured using the method of Owens and Belcher [37]. 100 μL of the sample or 100 mL of Tris-HCl buffer (50 mM, pH 7.4) was added to 1500 mL of DNTB and 500 mL of Tris-HCl (Sigma-Aldrich) buffer (50 mM, pH 7.4). The tubes were incubated for 60 min at room temperature and the absorbances were read against the blank at 412 nm. The GSH concentration was calculated using an extinction coefficient of 13,600 mol^−1^ cm^−1^. The concentration of GSH was expressed as mmol/g of protein in the tissue.

### 2.9. MDA Level

The quantity of MDA was determined using the method of Agbor and Odetola [38]. 250 μL of PBS or 20 mL of the sample was introduced in the control tube and in the test tubes, respectively. It has been added into each tubes, Tris-HCl buffer (250 mL, 50 mM, pH 7.4), trichloroacetic acid (500 mL, 20 % (*v*/*v*), Sigma-Aldrich), and thiobarbituric acid (100 mL, 0.67 %, Sigma-Aldrich). The mixture solution was heated in a water bath (90°C, 10 min). The tubes were then cooled in an ice bath and centrifuged (3000 rpm for 10 min). The supernatant was collected and its optical density was read using a spectrophotometer at 532 nm. The MDA concentration was calculated using an extinction coefficient of 1.56 × 10^5^ mmol^−1^ cm^−1^. MDA level was expressed in mmol/g of protein in the tissue.

### 2.10. NO Level

The method described by Fermor et al. [39] was used. In test tubes containing 100 μL of the sample or 100 μL of NaNO_2_ (blank and standard tubes) were introduced 400 μL of distilled water followed by 500 μL of Griess reagent. All tubes were then incubated in the dark for 10 min and the optical densities were read against the blank using a spectrophotometer at 546 nm. The concentration of NO content in the brain and spinal cord was expressed in μmol/g of the brain or spinal cord tissue.

### 2.11. GABA Concentration

This test is based on the coloration formed when GABA reacts with ninhydrin in an alkaline medium and in the presence of glutamate [40]. The homogenate supernatant was withdrawn to 100 μL while 1000 μL of GABA solution (100, 150, 200, 250, 300, 350, and 400 mg) was introduced into tubes. These tubes contained 200 μL of 0.14 M ninhydrin (prepared in carbonate–bicarbonate buffer [0.5 M, pH 9.9]) and 100 μL of 10% (*v*/*v*) concentrated glacial TCA. 100 μL of 0.015 mg/μL glutamate (prepared in 10% TCA) was introduced only into the tubes containing the different GABA solutions. The tubes were incubated at 60°C for 30 min and 5000 μL of copper tartrate (2.229 mg/mL) was introduced into each cooled tube. The tubes were incubated again at 25°C for 10 min and then cooled. 5 min later, the absorbance was read using a spectrophotometer at a wavelength of 451 nm against the blank. The concentration of GABA was determined by the measurement of the formed fluorescent product resulting from the reaction of GABA with ninhydrin an alkaline medium, in the presence of glutamate. The GABA content in the brain and spinal cord was expressed in mg/g of the brain or spinal cord tissue.

### 2.12. 5-HT Concentration

The concentration of 5-HT spinal cord was determined by the colorimetric assay of spinal cord homogenates as described by Schlumpf et al. [41]. In the presence of HCl, serotonin degrades to give a compound that reacts with OPT to give a chromophore. Briefly, 250 μL of crude HCl was introduced into a white tube. The homogenate samples were taken at 250 μL and introduced into dry tubes, and then 250 μL of OPT was added to these tubes. The whole mixture was boiled in a marine bath at 100°C for 10 min. After cooling, the absorbance was read at 470 nm. The 5-HT concentration was expressed in pg/mg of protein in the tissue.

### 2.13. Statistical Analysis

The individual values of each test were used to calculate the mean and the results were expressed as mean ± standard error of the mean (SEM) or standard deviation (SD). One-way analysis of variance followed by Tukey's test (behavioral tests) and two-way analysis of variance followed by Bonferroni's test (biochemical assays and *in vitro* tests) were used to compare group tests with controls (normal and/or negative). At *p* < 0.05, the differences were significant.

## 3. Results

### 3.1. Quantitative Phytochemistry Screening and LC-MS Analysis of *C. quadrangularis*

**Figure 1:**
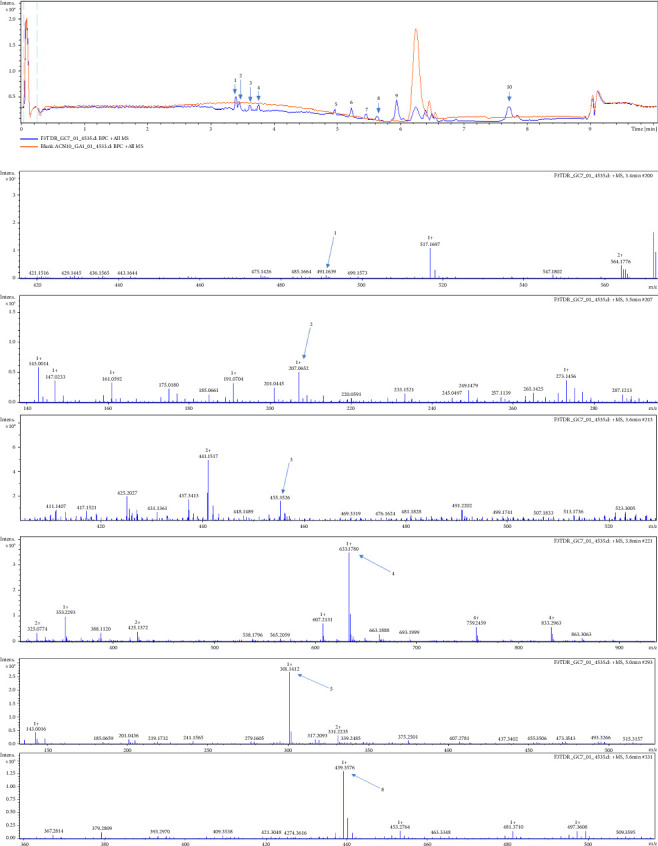
Total ion current (TIC) and extracted ion chromatograms of compounds.

The quantitative phytochemistry carried out on ethanolic extract of *C. quadrangularis* shows that the extract contains flavonoids, phenols, and tannins. The phenolic compounds, the flavonoids, and the tannins represent, respectively, 15.53 mg equivalent gallic acid/g of phenolic compounds, 2.55 mg equivalent of quercetin/g of flavonoids, and 8.02 mg equivalent of tannic acid/g of tannin (Table 1). The phytochemical analysis using LC-MS revealed the presence of 6 compounds (Figure 1 and Table 2): piperaduncin A, eugenitol, betulinic acid, paeoniflorin, angoletin, and momordicinin.

### 3.2. Effects of Extract on Some Behavioral Parameters of Rats Submitted to Vincristine-Induced Neuropathic Pain

**Figure 2:**
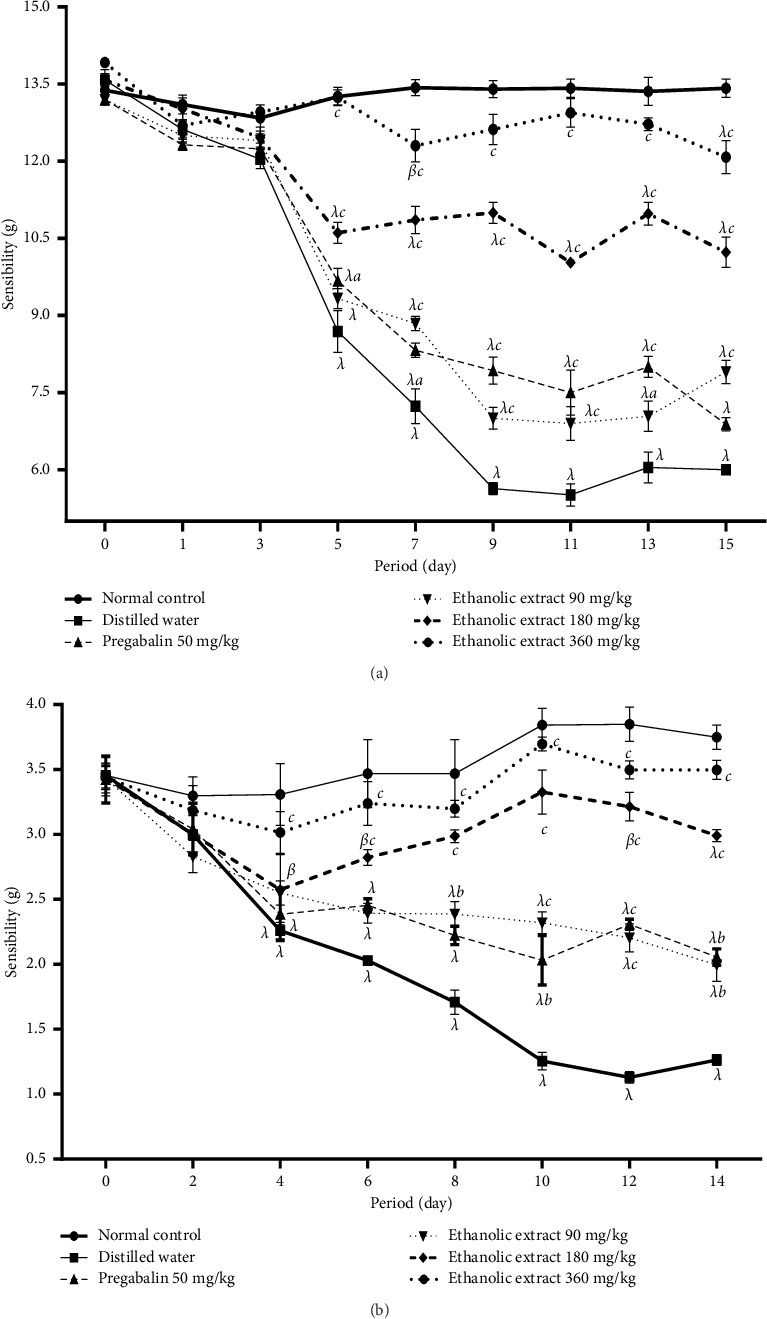
Effect of ethanolic extract of *Cissus quadrangularis* and pregabalin on paw hyperalgesia (a) and paw allodynia (b) in vincristine-induced neuropathic pain. Data are expressed as the mean ± SEM of six animals per experimental group, compared by two-way analysis of variance followed by Bonferroni's test. ⁣^*β*^*p* < 0.01 and ⁣^*λ*^*p* < 0.001 are significantly different compared to the normal control. ⁣^*a*^*p* < 0.05, ⁣^*b*^*p* < 0.01, and ⁣^*c*^*p* < 0.001 are significantly different compared to distilled water.

Figure 2 shows the effects of ethanolic extract of *C. quadrangularis* on mechanical hyperalgesia and mechanical allodynia. It emerges from these results that compared to the normal control group, after injection of vincristine, the animals of the negative control group gradually develop an increase in sensitivity to pain, reflecting the onset of peripheral neuropathy. This hypersensitivity, marked by a reduction in the pain sensitivity latency time, was significant (*p* < 0.001) from the fifth day for hyperalgesia and from the fourth day for allodynia in the animals of the negative control group. Oral administration of ethanolic extracts of *C. quadrangularis* or pregabalin protected animals against hypersensitivity and allodynia by increasing the latency time. For all the behavioral parameters studied, the effect of the ethanolic extract of *C. quadrangularis* (180 and 360 mg/kg) was significant (*p* < 0.01; *p* < 0.001) from Day 6 to Day 15, compared to the negative group control.

### 3.3. Effects of Ethanolic Extract on NO Level

**Figure 3:**
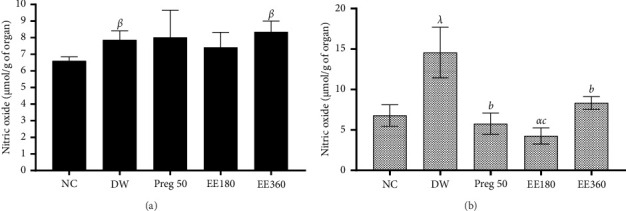
Effect of ethanolic extract of *Cissus quadrangularis* (EE) and pregabalin (preg) on NO level in the brain (a) and spinal cord (b) in vincristine-induced neuropathic pain. Data are expressed as the mean ± SEM of six animals per experimental group, compared by one-way analysis of variance followed by Tukey's test. ⁣^*α*^*p* < 0.05, ⁣^*β*^*p* < 0.01, and ⁣^*λ*^*p* < 0.001 are significantly different compared to the normal control. ⁣^*b*^*p* < 0.01 and ⁣^*c*^*p* < 0.001 are significantly different compared to distilled water.

Administration of vincristine resulted in a significant (*p* < 0.01; *p* < 0.001) increase in NO levels in the brain and spinal cord compared to the negative control. However, treatment with pregabalin (50 mg/kg) and ethanolic extract of *C. quadrangularis* (180 and 360 mg/kg) significantly (*p* < 0.01; *p* < 0.001) decreased the level of NO in the spinal cord compared to the negative control (Figure 3).

### 3.4. Effects of Ethanolic Extract on MDA Level and GSH Activity

**Figure 4:**
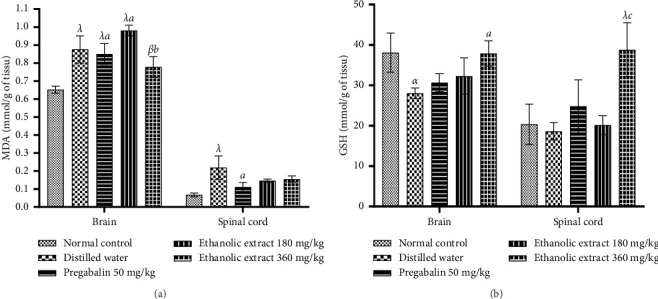
Effect of ethanolic extract of *Cissus quadrangularis* (EE) and pregabalin (preg) on MDA level (a) and GSH activity (b) in the brain and spinal cord in vincristine-induced neuropathic pain. Data are expressed as the mean ± SEM of six animals per experimental group, compared by one-way analysis of variance followed by Tukey's test. ⁣^*α*^*p* < 0.05 and ⁣^*λ*^*p* < 0.001 are significantly different compared to the normal control. ⁣^*a*^*p* < 0.05 and ⁣^*c*^*p* < 0.001 are significantly different compared to distilled water.

Figure 4 shows that vincristine injections resulted in a significant (*p* < 0.001) increase in the MDA level in the brain and spinal cord compared to the control group. Administration of the extract (180, 360 mg/kg) and pregabalin (50 mg/kg) led to a significant (*p* < 0.05) drop in the level of MDA in the spinal cord compared to the negative control group. However, in the brain, only the extract (360 mg/kg) resulted in a significant (*p* < 0.05) decrease in the MDA level compared to the negative control group.

Figure 4 also shows the effects of ethanolic extracts of *C. quadrangularis* on GHS activity in the brain and spinal cord. It was observed that the activity of GHS in the brain was significantly (*p* < 0.01) decreased in the animals of the negative control group compared to the animals of the normal control group. The treatment of the animals with the extract of *C. quadrangularis* (360 mg/kg) significantly (*p* < 0.05) increased the GHS activity in the brain and spinal cord compared to the negative control.

### 3.5. Effects of Extract on Total Calcium Level, GABA, and 5-HT Level

**Figure 5:**
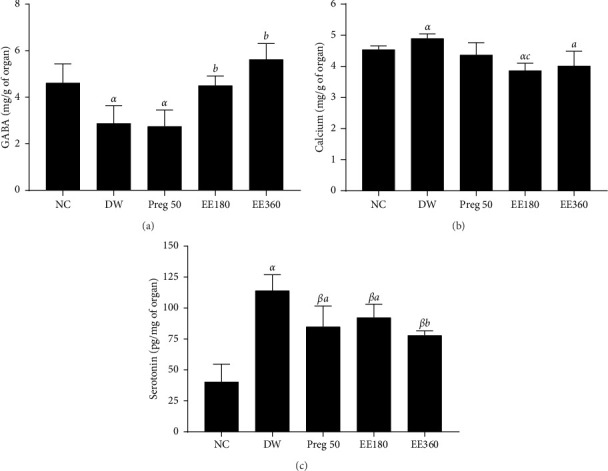
Effect of ethanolic extract of *Cissus quadrangularis* (EE) and pregabalin (preg) on GABA (a), calcium (b), and serotonin (c) levels in vincristine-induced neuropathic pain. Data are expressed as the mean ± SEM of six animals per experimental group, compared by one-way analysis of variance followed by Tukey's test. ⁣^*α*^*p* < 0.05 and ⁣^*β*^*p* < 0.01 are significantly different compared to the normal control. ⁣^*a*^*p* < 0.05, ⁣^*b*^*p* < 0.01, and ⁣^*c*^*p* < 0.001 are significantly different compared to distilled water.

Figure 5 shows that the injection of vincristine resulted in a significant (*p* < 0.05) increase in the total calcium level in the sciatic nerve compared to the normal control. The treatment of animals with ethanolic extract of *C. quadrangularis* (180 and 360 mg/kg) significantly (*p* < 0.05; *p* < 0.001) reduced the total calcium level compared to the negative control group. Vincristine injections significantly (*p* < 0.05) reduced GABA levels in the spinal cord of animals in the negative control group compared to the control group. Administration of the extract resulted in a significant (*p* < 0.01) increase in GABA levels in the spinal cord compared to the negative control group (Figure 5). The 5-HT level increased significantly (*p* < 0.001) in animals in the negative control group compared to those in the normal control group. Administration of pregabalin (50 mg/kg) and extract (180 and 360 mg/kg) resulted in a significant (*p* < 0.05; *p* < 0.01) decrease in 5-HT level compared to the negative control group.

## 4. Discussion

This study was undertaken with the aim of evaluating *in vivo* the antihyperalgesic properties (hyperalgesia and allodynia) of ethanolic extract of *C. quadrangularis*. It appears from this study that the ethanolic extract of this plant significantly inhibits mechanical hyperalgesia and allodynia after vincristine injection. This extract significantly reduces the levels of NO, MDA, calcium, and serotonin, and then increases the concentration of GSH and GABA.

Neuropathic pain, which is a consequence of damage to the nervous system that can induce hypersensitivity (hyperalgesia and/or allodynia), remains a very difficult pathology to treat today [42]; thus, a simple sensation of touching or accidently rubbing door frames or furniture (allodynia) or a pat on the back (hyperalgesia) becomes very painful [43]. Cancer chemotherapeutic agents, such as vincristine are known to induce peripheral neuropathic pain through the destruction of the blood–nerve barrier [44]. Allodynia and hyperalgesia are the main symptoms of peripheral neuropathy [45].

In this study, *Wistar* rats treated with the intraperitoneal injection of vincristine developed mechanical hyperalgesia and allodynia which are the major symptoms of neuropathic pain [46], and the use of analgesimeter (hyperalgesia) and Von Frey filaments (allodynia) for the evaluation of antinociceptive substances is widely adopted [47]. People (38%–100%) with cancer receiving chemotherapy treatment with anticancer drugs (paclitaxel, vincristine, and docetaxel) are subjected to peripheral neuropathy with allodynia and hyperalgesia as the main symptoms [48], which in most cases are resistant to effective analgesics on other pain models [49]. This will affect the survival and quality of life of cancer patients [50]. This study showed that *C. quadrangularis* extracts are effective in reversing hyperalgesic and allodynic symptoms induced by vincristine injection. Interestingly, pretreatment with *C. quadrangularis* extracts prevented *Wistar* rats from developing hyperalgesia and allodynia, indicating that pretreatment with the extracts attenuates neuronal plasticity that could have resulted in hyperalgesia and allodynia. The results of this study suggest that the extracts possess an antihyperalgesic effect similar to that of pregabalin, an anticonvulsant drug.

Cancer drugs can cause nerve damage by damaging nuclear and/or mitochondrial DNA, altering axonal transport, disrupting ion channels, causing loss of antioxidant enzymes, altering the inflammatory process, and increasing oxidative stress [50]. The overproduction of ROS during neuropathic pain, evidenced by an increase in markers of oxidative stress (DNA oxidation and lipid peroxidation) justifies the reduction in antioxidant defense [51]; this incipient oxidative stress causes on the one hand the damage of non-neuronal and neuronal cells and on the other hand the activation of macrophages with overproduction of proinflammatory substances [52]. Several studies evidenced that the oxidative stress and accumulation of calcium in nerves play a crucial role in the pathogenesis of peripheral neuropathic pain caused by chemotherapy [53]. In addition, it has been reported that the accumulation of calcium in the nerve leads to oxidative stress in the central nerve system and alteration in the pain modulation system [54]. This study shows that vincristine administration induced an increase in total calcium content and serotonin in the sciatic nerve, modified oxidative stress parameters (an increase in MDA and a decrease in GSH activity) in the brain and spinal cord, and reduced GABA levels in the spinal cord. In fact, it has been reported that a drop in the GABA level in the spinal cord will often lead to neuronal hyperexcitability and contributes to the maintenance of neuropathic pain [55]. In addition, it has been reported that during the development of neuropathic pain, the expression of 5-HT3 receptors is upregulated, which also contributes to the maintenance of this pain [56]. In our investigations, administration of ethanolic extract of *C. quadrangularis* has significantly attenuated the previously reported biochemical changes associated with vincristine-induced neuropathic pain. Indeed, the administration of the ethanolic extract of this plant significantly increases the concentration of NO in the spinal cord; this extract significantly reduced the level of MDA and significantly increased the concentration of GSH in the brain and spinal cord. Moreover, the same extract significantly reduced the concentration of GABA, calcium, and serotonin.

In fact, *C. quadrangularis* is reported to exert various beneficial pharmacological actions in conditions such as epilepsy, depression, and cell proliferation [57]. Indeed, it has also been reported that this plant reveals the presence of terpenoids, flavonoids, alkaloids, glycosides, cardiac glycosides, saponins, and tannins [58]. Moreover, many biologically active compounds have already been identified from the ethanolic extract of *C. quadrangularis* in this study, and these include piperaduncin A, eugenitol, betulinic acid, paeoniflorin, and angoletin. Furthermore, it has also been reported that betulinic acid and angoletin have antioxidant properties [59]; betulinic acid is able to inhibit calcium influx in dorsal root ganglion neurons by inhibiting T- (CaV3) and N- (CaV2.2) type voltage-gated calcium channels, and this effect leads to the inhibition of depolarization-evoked calcium influx in dorsal root ganglion (DRG) [60]; in addition, betulinic acid reversed mechanical allodynia in rat models of chemotherapy-induced peripheral neuropathy and HIV-associated peripheral sensory neuropathy as well as in the mouse model of partial sciatic nerve ligation [61]. It is known that paeoniflorin could significantly alleviate Cuff-induced hyperalgesia and depressive behaviors, lessen the pathological damage to the hippocampal cell, reduce proinflammatory cytokine levels, and inhibit microglial overactivation. Furthermore, paeoniflorin downregulated the expression levels of TLR4/NF-*κ*B signaling pathway–related proteins in the hippocampus [62]. Paeoniflorin has antioxidant properties, GABA agonist properties, neuroinflammation inhibitor properties, intracellular calcium increase inhibitor properties and opioid receptor agonist properties [63]. Therefore, attenuation of hyperalgesia, allodynia, and associated biochemical parameters caused by vincristine injections may be possible by the combination of different properties of the compounds contained in the ethanolic extract. These results suggest that treatment with ethanolic extract of this plant may play an important role in the regulation of pain at central and peripheral levels [64].

## 5. Conclusions

The *in vivo* antihyperalgesic properties of ethanolic extract of *C. quadrangulari*s were demonstrated in this study. Extract of *C. quadrangularis* also induced a significant reduction of hyperalgesia and allodynia observed after vincristine injection. The results of this study suggest that extracts of *C. quadrangularis* use antioxidative activity, calcium inhibitory activity, and neuromodulation capacity to prevent the development of painful neuropathy after vincristine administration. This demonstrates that *C. quadrangularis* is a promising molecule for the management of peripheral neuropathic pain induced by anticancer drugs.

## Figures and Tables

**Table 1:** Total phenol, flavonoid, and tannin content of ethanolic extracts of *Cissus quadrangularis*.

	Total phenol (mg gallic acid eq/mg extract)	Total flavonoid (mg equivalent of quercetin/mg extract)	Total tannin (mg equivalent of tannic acid/g extract)
EA	15.57 ± 0.16	2.07 ± 0.33	1.48 ± 0.62
Butylhydroxytoluène	426.85 ± 0.68	61.89 ± 0.97	52.43 ± 0.31

**Table 2:** Main signals exhibited in the LC-MS spectra of compounds detected in the ethanolic extract of *Cissus quadrangularis* and proposed attribution.

No	Tr (min)	[M + H]^+^		Err (ppm)	Molecular formula	Name of compound
		Exp	Calcl			
1	3.4	491.2095	491.2064	−6.2	C_29_H_30_O_7_	Piperaduncin A
2	3.5	207.0649	207.0652	1.6	C_11_H_10_O_4_	Eugenitol
3	3.6	455.3526	455.3520	−0.6	C_30_H_46_O_3_	Betulinic acid
4	3.8	633.1784	633.1814	4.8	C_30_H_32_O_15_	Paeoniflorin
5	5.0	301.1412	301.1434	7.3	C_18_H_20_O_4_	Angoletin
6	5.2	377.2668	377.2686	4.9	C_23_H_36_O_4_	Not identified
7	5.4	379.2819	379.2843	6.2	C_23_H_38_O_4_	Not identified
8	5.6	439.3577	439.3571	−1.4	C_30_H_46_O_2_	Momordicinin
9	5.9	413.2668	413.2686	4.3	C_26_H_37_O_4_	Not identified
10	7.7	871.5734	871.5719	−1.7	C_54_H_79_O_9_	Not identified

## Data Availability

The data that support the findings of this study are available from the corresponding authors on request.

## Nomenclature

*C. quadrangularis*: *Cissus quadrangularis*
GABA: Gamma-aminobutyric acid
5-HT: Serotonin
MDA: Malondialdehyde
GSH: Glutathione
NF-*κ*B: Nuclear factor kappa B
H_3_PW_12_O_40_: Phosphotungstic acid
H_3_PMo_12_O_40_: Phosphomolybdic acid
HCl: Chloridric acid
OPT: O-phthaldialdehyde
TCA: Trichloroacetic acid
DAD: Diode array detector
PBS: Phosphate buffered saline
NO: Nitrite oxyde
Ca^2+^: Calcium ion
SEM: Standard error of the mean
SD: Standard deviation
ROS: Reactive oxygen species
DRG: Dorsal root ganglion
